# An Eocene paraclupeid fish (Teleostei, Ellimmichthyiformes) from Bolca, Italy: the youngest marine record of double‐armoured herrings

**DOI:** 10.1002/spp2.1230

**Published:** 2018-07-30

**Authors:** Giuseppe Marramà, Alexandre F. Bannikov, Jürgen Kriwet, Giorgio Carnevale

**Affiliations:** ^1^ Department of Palaeontology University of Vienna Althanstrasse 14 1090 Vienna Austria; ^2^ Borisyak Paleontological Institute of the Russian Academy of Sciences Profsoyuznaya 123 Moscow 117647 Russia; ^3^ Dipartimento di Scienze della Terra Università degli Studi di Torino via Valperga Caluso 35 10125 Torino Italy

**Keywords:** *Eoellimmichthys superstes*, Teleostei, Ellimmichthyiformes, Paracluepidae, Bolca, Eocene

## Abstract

A new double‐armoured herring of the clupeomorph order Ellimmichthyiformes, *Eoellimmichthys superstes* gen. et sp. nov., is described herein based on a single partially complete specimen from the early Eocene Pesciara site of the Bolca Konservat‐Lagerstätte, north‐eastern Italy. The fossil documented herein exhibits a unique combination of features (e.g. ornamentation of the skull bones; medial fusion of the contralateral halves of the neural arches of abdominal vertebrae; teeth on endopterygoid, parhypural fused to the first preural centrum; presence of a short series of 6 (or 7) predorsal scutes increasing in size posteriorly; postpelvic scutes bearing very prominent spines), which clearly supports its recognition as a new genus and species of the family Paraclupeidae. Evidence is provided to show that *Eoellimmichthys* gen. nov. is most closely related phylogenetically to the Early Cretaceous genus *Ellimmichthys*. The new taxon described herein represents the youngest marine occurrence of the family Paraclupeidae and, more generally, of the order Ellimmichthyiformes, suggesting that the shallow marine settings of the Tethys might have favoured the persistence of certain fish lineages that were severely affected by the end‐Cretaceous mass extinction.

Double‐armoured herrings of the order Ellimmichthyiformes constitute an extinct clade of clupeomorph fishes characterized by a remarkably wide palaeogeographical and palaeoenvironmental distribution in marine, estuarine and freshwater deposits of Africa, Asia, Europe, and North and South America (see e.g. Grande 1982, 1985; Chang & Maisey 2003; Forey *et al*. 2003; Murray & Wilson 2013; Bannikov 2015; Vernygora & Murray 2015; Marramà & Carnevale 2017a). This clupeomorph clade currently includes about 40 species arranged in 19 genera spanning from the Early Cretaceous to the middle Eocene (e.g. Grande 1982; Alvarado‐Ortega & Melgarejo‐Damián 2017; Figueiredo & Ribeiro 2017). However, the diversity of this extinct lineage can be easily increased with the revision of a number of species traditionally assigned to the genera ‘*Clupea*’ or ‘*Diplomystus*’ (Grande 1985). After what is possibly their first appearance in the Early Cretaceous, the Ellimmichthyiformes experienced a proliferation of new anatomies during the Late Cretaceous (mostly Cenomanian; see Forey *et al*. 2003; Khalloufi *et al*. 2010) and, probably, the exploitation of new ecological strategies. After the end‐Cretaceous mass extinction and until their final extinction in the middle Eocene, the representatives of the Ellimmichthyiformes experienced a drastic drop in their diversity and disparity, with the last Palaeogene taxa being restricted to the freshwaters of North and South America, and China (Grande 1982; Chang & Maisey 2003; Marramà & Carnevale 2017a), providing further support for the hypothesis that continental waters suffered lower proportionate extinctions compared to marine environments (Robertson *et al*. 2013).

The celebrated upper Ypresian (*c*. 49 Ma) marine deposits of the Bolca Konservat‐Lagerstätte have yielded an enormous number of exquisitely preserved fishes that provide clear evidence of the highly successful early Palaeogene fish radiation in the aftermath of the end‐Cretaceous extinction (Marramà *et al*. 2016a, b). Although about 250 species of bony and cartilaginous fishes (e.g. Blot 1987; Blot & Tyler 1990; Bannikov 2004, 2006, 2008; Monsch 2006; Bannikov & Carnevale 2009, 2010, 2016; Carnevale & Pietsch 2009, 2010, 2011, 2012; Carnevale *et al*. 2014, 2017; Marramà & Carnevale 2017b; Marramà *et al*. 2017a, 2017b, 2018b) have been described from this locality so far, double‐armoured herrings have not previously been reported. On the other hand, clupeomorph fishes are by far the most common group of fishes in this deposit, represented by four taxa belonging to the order Clupeiformes: the sardine *Bolcaichthys catopygopterus*, the round herring *Trollichthys bolcensis*, the possible shad *Eoalosa janvieri*, and the anchovy *Eoengraulis fasoloi* (2015a, 2015b, 2016, 2018). A recent re‐examination of the historical material from the Pesciara site has revealed the presence of a new clupeomorph represented by a single specimen whose diagnostic characters unquestionably support its assignment to the order Ellimmichthyiformes, therefore representing the first record of the double‐armoured herrings in the Bolca fish assemblage and, at the same time, the youngest occurrence of this clade in a marine palaeobiotope.

## Geological setting

**Figure 1:**
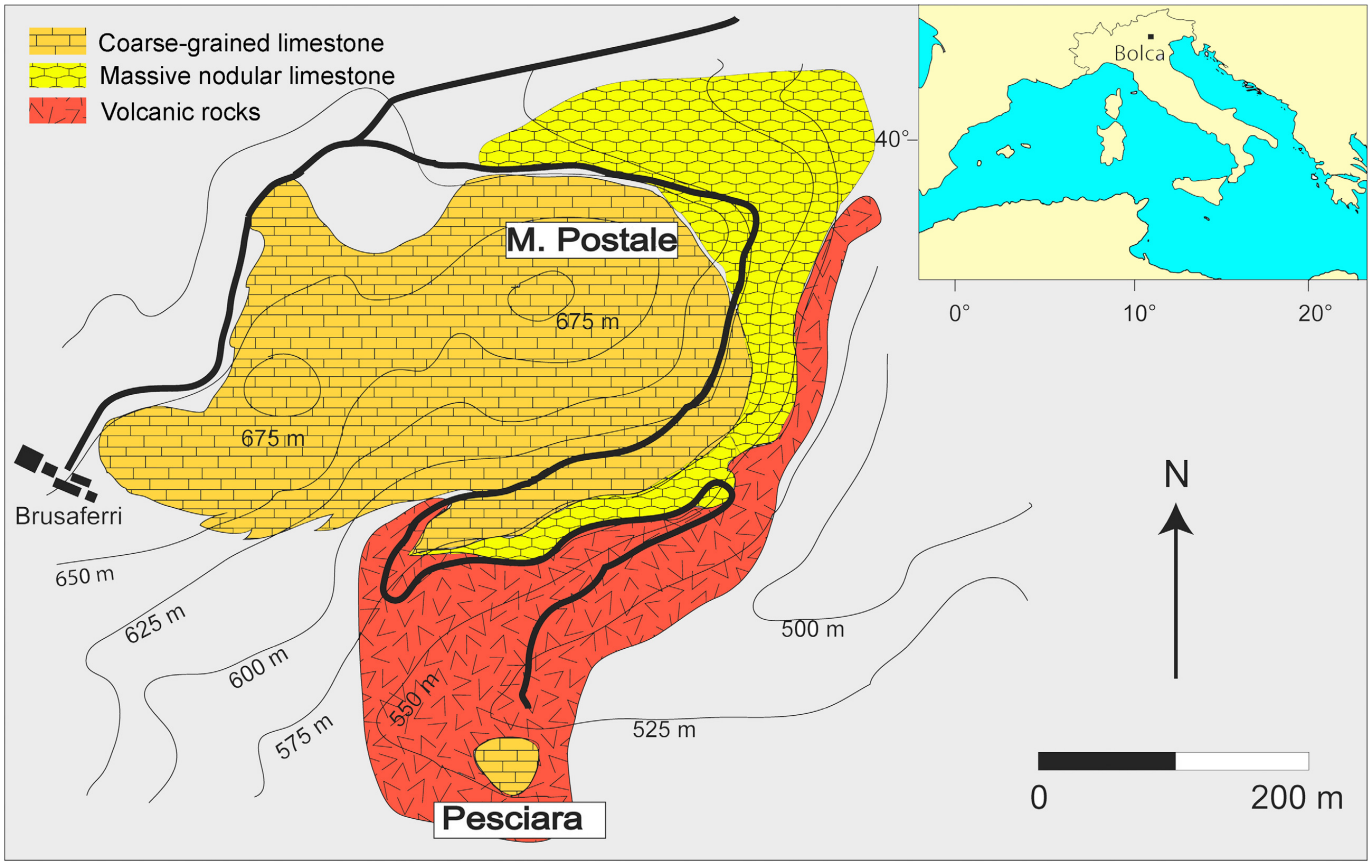
Location and geological map of the Bolca area showing the Pesciara site of Ypresian age where *Eoellimmichthys superstes* gen. et sp. nov. has been found. Adapted and modified from Trevisani (2015) and Marramà *et al*. (2018b). Colour online.

The specimen described herein comes from the fossiliferous strata of the Pesciara, one of the main productive sites of the Bolca Konservat‐Lagerstätte, north‐eastern Italy (Fig. 1). The calcareous sequence of the Pesciara site has been traditionally referred to as ‘Calcari Nummulitici’, an informal unit of Eocene age widely distributed in north‐eastern Italy (Papazzoni & Trevisani 2006). This succession consists of a cyclic alternation of about 20 m of finely laminated micritic limestones (containing exquisitely well‐preserved fishes, plants and invertebrates) and coarse‐grained biocalcarenite/biocalcirudite with a rich benthic fauna. Based on their larger benthic foraminiferan content, the fish‐bearing limestones of the Pesciara site were referred to the *Alveolina dainelli* Zone, corresponding to the late Cuisian (late Ypresian, slightly less than 49 Ma; Papazzoni & Trevisani 2006; Papazzoni *et al*. 2014). The results of a recent quantitative palaeoecological analysis by Marramà *et al*. (2016c) suggested that the Pesciara fish assemblage was characterized by a sharp oligarchic structure dominated by planktivorous fishes (mostly clupeoids), whereas the sedimentological and taphonomic features concur to suggest that the fossiliferous sediments accumulated in a shallow intraplatform basin in which anoxic conditions and the development of a biofilm at the bottom promoted the high‐quality preservation of the fossils (see also Papazzoni & Trevisani 2006).

## Material and method

The present study is based on a single well‐preserved specimen whose preservation quality and slab lithology clearly indicate that it was collected from the Pesciara site. The specimen, MCSNV IG.23695, is currently housed in the Museo Civico di Storia Naturale di Verona, Italy. The fossil was examined using a Leica M80 stereomicroscope equipped with camera lucida drawing arm, and alcohol was used to enhance some details of its skeletal anatomy. UV light was used in an attempt to enhance the visibility of some of the skeletal structures. However, the use of this technique was unhelpful in this case (see Marramà *et al*. 2018a) since the bones of certain fishes of the Pesciara site do not reflect any fluorescence (R. Zorzin, pers. comm. April 2018). Measurements were taken with a dial calliper to the nearest 0.1 mm. Standard length (SL) is used throughout. Osteological terminology follows primarily Murray & Wilson (2013), Vernygora & Murray (2015) and Marramà & Carnevale (2017a). Comparative information is mostly derived from the literature.

The phylogenetic analysis is based on that carried out in the recent study by Marramà & Carnevale (2017a), which in turn is based on the morphological datasets of Alvarado‐Ortega *et al*. (2008), Murray & Wilson (2013) and Vernygora & Murray (2015). The data matrix contains 62 unordered and unweighted characters taken from the above‐mentioned cladistic analyses (Marramà *et al*. 2018a). The characters were imported into Mesquite 3.03 (Maddison & Maddison 2008) and the data for the new taxon were added. The phylogenetic analysis was performed with TNT 1.5 (Goloboff *et al*. 2008), using the branch‐and‐bound method with tree bisection reconnection (TBR) algorithm, and collapsing threes after search. Tree length, consistency (CI) and retention (RI) indices were then calculated for the strict consensus tree.

## Systematic palaeontology

### Superorder CLUPEOMORPHA Greenwood *et al*., 1966 Order ELLIMMICHTHYIFORMES Grande, 1982 Family PARACLUPEIDAE Chang & Chou, 1977 Genus EOELLIMMICHTHYS nov.

#### LSID

urn:lsid:zoobank.org:act:C99A9056‐3D91‐4A06‐BE1A‐AB3E2F340F1A

#### Type species

*Eoellimmichthys superstes* sp. nov.

#### Derivation of name

The name is derived from the Greek *Ēōs*, pertaining to the dawn or sunrise, as well as to the goddess of dawn, corresponding to the Latin *Aurora*; and *Ellimmichthys*, indicating a close relationship of this latter genus with the new taxon; hence an ‘ellimmichthys‐like fish from the Eocene’; gender masculine.

#### Other species included

Type species only.

#### Diagnosis

Deep‐bodied paraclupeid fish showing the following combination of characters: head large, about 40% SL; maximum body depth about 60% SL; anterior dorsal margin of the body almost straight, forming a marked angle at the dorsal‐fin insertion; posttemporal sharp and slender; cleithrum S‐shaped; epineurals and epipleurals absent in the caudal region; 31 (12 + 19) preural vertebrae; ten pairs of ribs; about 16 dorsal and 25 anal‐fin rays supported by an equal number of pterygiophores; six supraneurals; complete series of 6 (or 7) predorsal scutes; anteriormost scutes small, not significantly broader than long; posteriormost scutes larger and bearing a strong and prominent median posteriorly‐directed spine; postdorsal scutes absent; complete series of 12 abdominal scutes (six prepelvic and six postpelvic) bearing a prominent ventral posteriorly‐directed spine; abdominal scute wings spine‐like, with large spaces between wings of adjacent scutes.

#### Remarks

The holotype specimen (MCSNV IG.23695) described herein was included in the list of the material studied by Blot (1987, p. 16) in his work devoted to the study of the pycnodontiform fishes from Bolca, and referred to as a small individual of the species *Pycnodus platessus*. A recent examination of the material formerly reported by Blot (1987) quickly revealed that MCSNV IG.23695 does not show any of the pycnodont diagnostic features; rather it shows several diagnostic characters of the double‐armoured herrings that support its attribution to this group of extinct clupeomorph fishes.

## *Eoellimmichthys superstes* sp. nov. Figures 2, 3, 4, 5

**Figure 2:**
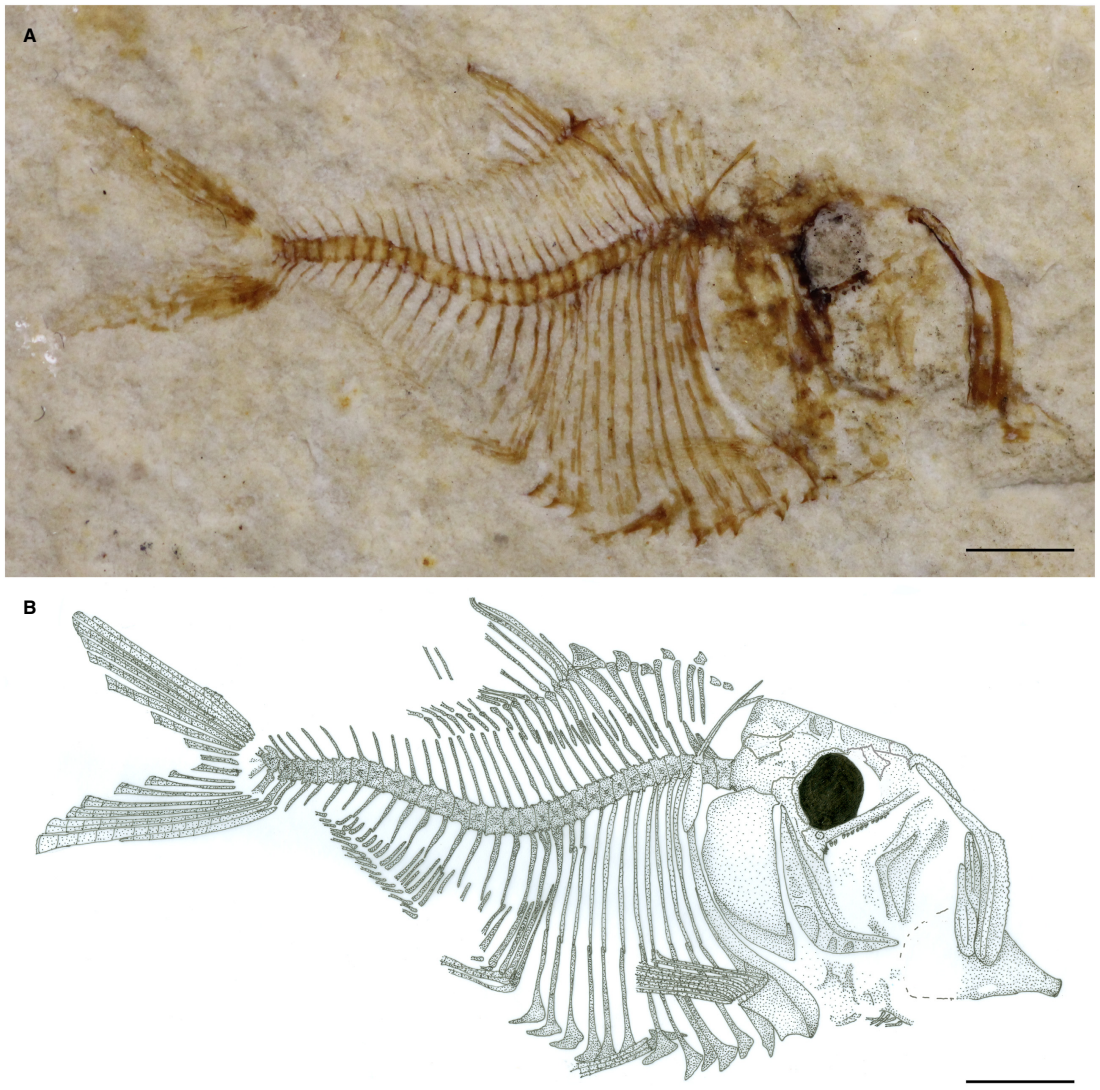
*Eoellimmichthys superstes* gen. et sp. nov. from the early Eocene of Bolca. A, MCSNV IG.23695, holotype, right lateral view. B, interpretative reconstruction. Scale bars represent 2 mm. Colour online.

**Figure 3:**
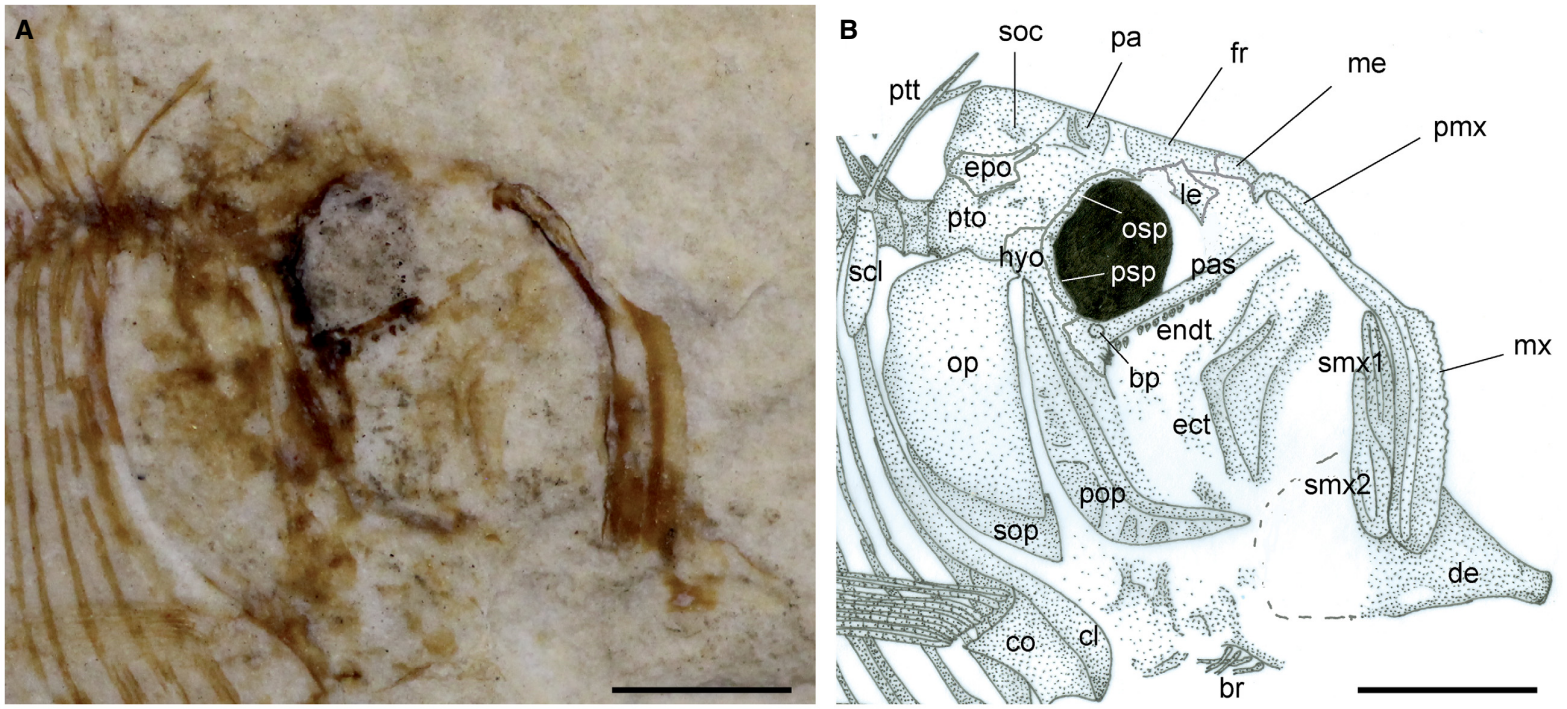
*Eoellimmichthys superstes* gen. et sp. nov. from the early Eocene of Bolca. A, MCSNV IG.23695, holotype, detail of the cranial region and pectoral girdle, right lateral view. B, interpretative reconstruction. *Abbreviations*: bp, ‘basipterygoid’ process; br, branchiostegal rays; cl, cleithrum; co, coracoid; de, dentary; ect, ectopterygoid; endt, endopterygoid teeth; epo, epioccipital; fr, frontal; hyo, hyomandibula; le, lateral ethmoid; me, mesethmoid; mx, maxilla; op, opercle; osp, orbitosphenoid; pa, parietal; pas, parasphenoid; pmx, premaxilla; pop, preopercle; ptt, posttemporal; psp, pterosphenoid; pto, pterotic; scl, supracleithrum; smx, supramaxilla; soc, supraoccipital; sop, subopercle. Scale bars represent 2 mm. Colour online.

**Figure 4:**
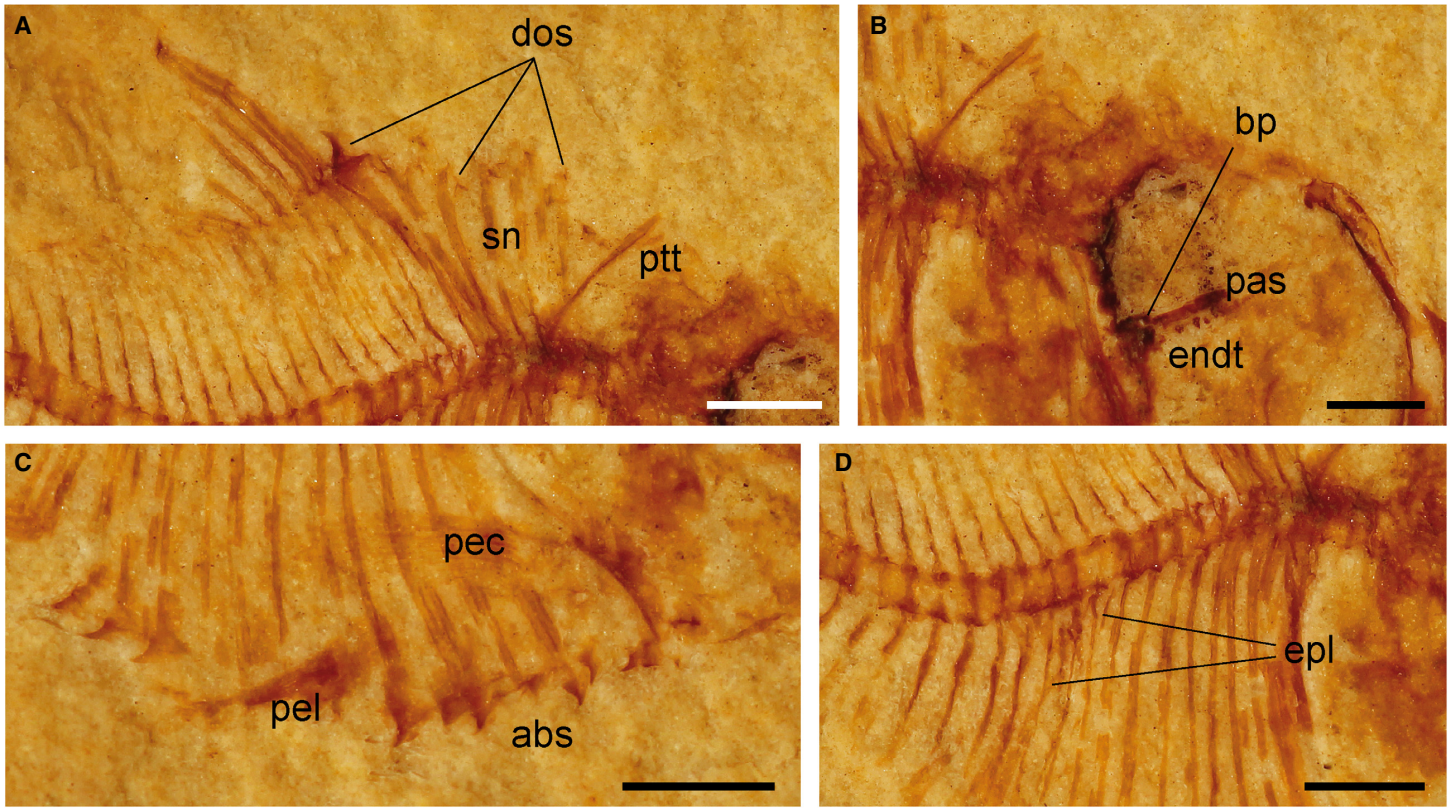
*Eoellimmichthys superstes* gen. et sp. nov. from the early Eocene of Bolca; MCSNV IG.23695, holotype, right lateral view. A, close‐up of the dorsal fin and predorsal margin of the body. B, close‐up of the orbital region. C, detail of the ventral margin of the abdominal region. D, close up of the intermuscular bones. *Abbreviations*: abs, abdominal scutes; bp, ‘basipterygoid’ process; dos, predorsal scutes; endt, endopterygoid teeth; epl, epipleurals; pas, parasphenoid; pec, pectoral fin; pel, pelvic fin; ptt, posttemporal; sn, supraneurals. All scale bars represent 1 mm. Colour online.

**Figure 5:**
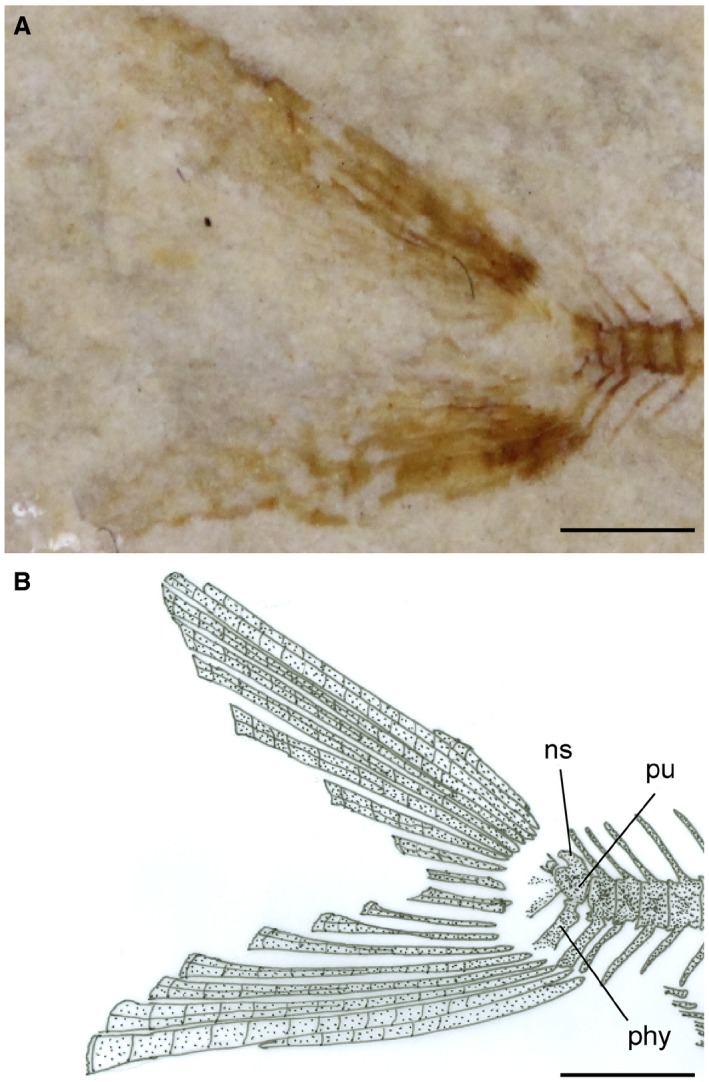
*Eoellimmichthys superstes* gen. et sp. nov. from the early Eocene of Bolca. A, MCSNV IG.23695, holotype, detail of the caudal region, right lateral view. B, interpretative reconstruction. *Abbreviations*: ns, neural spine; phy, parhypural; pu, first preural centrum. Scale bars represent 1 mm. Colour online.

### LSID

urn:lsid:zoobank.org:act:E8FF9E84‐4438‐40D8‐A70E‐BCC830822A78

### Derivation of name

The name is derived from the Latin *superstes* meaning ‘survivor’; in reference to its status as last marine survivor of the ellimmichthyiform clade.

### Type specimen

MCSNV IG.23695, holotype; well‐preserved, partially complete articulated skeleton; 13.7 mm SL (Fig. 2).

### Diagnosis

As for the genus.

### Measurements

All in mm with percentage of SL in parentheses: total length: 17.5 mm (127.7%); head length: 5.3 mm (38.7%); head depth: 6.5 mm (47.5%); preorbital length: 1.2 mm (8.8%); postorbital length: 2.4 mm (17.5%); orbit diameter: 1.6 mm (11.7%); maximum body depth: 8.3 mm (60.6%); caudal peduncle length: 1.4 mm (10.2%); caudal peduncle depth: 1.4 mm (10.2%); predorsal length: 7.4 mm (54.0%); preanal length: 9.4 mm (68.6%); prepectoral length: 5.2 mm (38.0%); prepelvic length: 7.7 mm (56.2%); dorsal‐fin base length: 3.1 mm (22.6%); anal‐fin base length: 4.9 mm (35.8%).

### Occurrence

Pesciara site, Bolca Lagerstätte, north‐eastern Italy; late early Eocene, late Ypresian, middle Cuisian, slightly less than 49 Ma (Papazzoni *et al*. 2014).

## Description

The specimen consists of a small sized fish, measuring about 14 mm SL. However, the considerable degree ossification of the skeleton seems to suggest that the specimen most likely represents a late juvenile or even a subadult individual. The body is laterally compressed, relatively deep, with a prominent and convex abdomen (Fig. 2). The maximum body depth measured at mid‐length between the dorsal fin and the posterior border of the neurocranium exceeds the half of SL. The head is large, deep, higher than long; its length is contained about 2.6 times in SL (Fig. 3). The snout is blunt and the mouth is large and terminal. The anterior dorsal margin of the body, from the posterior border of neurocranium to the dorsal‐fin origin, is almost straight, forming a marked angle at the dorsal‐fin origin. The belly is entirely covered with a complete series of abdominal scutes extending from the isthmus to the anal‐fin origin. Both the dorsal‐ and anal‐fin origins are located in the posterior half of the body, whereas the pelvic fins insert anteriorly to the dorsal‐fin origin. Squamation is not preserved.

### Cranium

The frontals are large, subtriangular and posteriorly expanded, covering about 50% of the length of the skull roof (Fig. 3). The parietals are small, subrectangular in shape and articulate anteriorly with the posterior margin of the frontals. Frontals and parietals appear to be ornamented by strong grooves. Although it is unclear if the parietals are separated from each other by the supraoccipital, it is likely that *Eoellimmichthys superstes* showed the condition of all ellimmichthyiforms in which the parietals articulate medially with each other (Alvarado‐Ortega *et al*. 2008; Murray & Wilson 2013). The supraoccipital crest is wide and subtriangular in lateral view. A distinct angle formed by the supraoccipital crest and the rest of the skull roof is not present, so that the lateral profile of the cranial roof appears straight from the anterior part of the frontal to the posterior end of the skull. The epioccipital is robust and contacts laterally the supraoccipital and anteriorly the parietal. The morphology of the posttemporal fossa is difficult to determine due to inadequate preservation of the temporal and otic regions of the skull. The orbitosphenoid and pterosphenoid are robust, curved, and form the dorsal and posterior walls of the orbit, respectively. The orbit is large, ovoid in shape and represents about 12% and 30% of SL and head length, respectively. Traces of the eyeball are preserved as a thin carbonaceous film (Figs 2, 3). The parasphenoid is robust and straight and occupies most of the basicranial length. Part of the ‘basipterygoid’ process can be recognized in the posterior part of the parasphenoid (Figs 3, 4B) resembling the condition figured by Grande (1982, fig. 6) in certain ellimmichthyiforms. The presence of a ‘osteoglossoid’ tooth patch is unclear. The vomer is not preserved. The mesethmoid is small and seems to articulate anteriorly with the premaxilla. The lateral ethmoid is small and articulates dorsally with the antero‐ventral margin of the frontal.

### Circumorbital series

The bones of the circumorbital series are inadequately preserved in the available specimen. Infraorbitals and nasals are not recognizable, as are the supraorbitals and the antorbital.

### Jaws

The mouth is large and terminal (Figs 2 and 3). Not surprisingly, the specimen shows an extremely gaping mouth, a clear indication of tetany, which is common in fishes from the Pesciara site (Marramà *et al*. 2016c). The premaxilla is long, narrow and slightly curved. It is considerably shorter than the maxilla and seems to possess a toothed oral margin. The maxilla is very large, elongate and laterally flattened. The maxilla is angled in the middle with a narrow anterior and an expanded posterior branch approximately of the same length and forming an angle of about 130° with each other. Small numerous teeth can be recognized along the oral margin of the maxilla. There are two supramaxillae; the first one is elongate and ovoid in shape, whereas the second one is slightly larger and paddle‐shaped. Only the anterior portion of the lower jaw is partially preserved. The dentary appears to be robust and deep, possibly trapezoid in shape, with an almost straight anterior margin. The articular–quadrate joint is not clearly recognizable but was probably located under the antero‐ventral border of the orbit.

### Suspensorium

Most of the bones of the suspensorium (metapterygoid, quadrate) are poorly preserved (Fig. 3). The endopterygoid appears to be nearly straight and antero‐posteriorly elongated; medially, it bears about 8–9 conical teeth arranged into a single row; the teeth increase in size posteriorly in the series (Fig. 4B). The hyomandibula is partially hidden by the opercular apparatus; however, it is possible to recognize its robust articular head.

### Opercular series and hyoid apparatus

The preopercle is crescent‐shaped, with the two arms forming an angle of about 125°; both arms are narrow, with the vertical one longer than the horizontal one. At least three branches of the preopercular sensory canal can be recognized (Figs 2, 3). The opercle is large, dorso‐ventrally elongated, subquadrangular in shape, with a rounded posterior margin; its outer surface appears to be smooth. The subopercle is small and subtriangular in shape, whereas the outline of the interopercle is unclear.

The bones of the hyoid apparatus are poorly preserved. Four incomplete branchiostegal rays are recognizable, although their number was certainly higher in origin.

### Vertebral column and intermuscular bones

The vertebral column consists of 31 preural vertebrae, of which 12 are abdominal (Fig. 2). The vertebral centra are subrectangular in shape, higher than long. The neural and haemal arches are well developed throughout the entire length of the vertebral column. The left and right halves of the anterior‐most neural spines appears to be fused to each other, as in several paraclupeid species (Alvarado‐Ortega *et al*. 2008; Murray & Wilson 2013). There are ten pairs of pleural ribs; the anteriormost ribs articulate with pits located along the lateral surface of the centra, whereas succeeding ones articulate with the parapophyses. Weak traces of epineurals and epipleurals can be recognized in the anterior region of the body (Fig. 4D), but they are clearly absent in the caudal region. The presence of epicentrals cannot be determined.

There are six robust and elongate supraneurals with a dorsal end reaching the dorsal border of the body (Fig. 4A); the supraneurals are slightly inclined posteriorly, lacking the fan arrangement characteristic of *Diplomystus* (Grande 1982; Murray *et al*. 2016).

### Caudal skeleton

The caudal skeleton is poorly preserved in the single available specimen (Fig. 5). The first preural centrum is separated from the first ural centrum. The neural spine of the first preural centrum is short and subrectangular. The number and structure of the poorly ossified hypurals is difficult to determine, although they probably might have been six in origin. The parhypural appears to be fused to the first preural centrum. The number and arrangement of uroneurals and epurals is difficult to recognize. The morphology of the caudal scutes cannot be recognized. The caudal fin is deeply forked and contains about 17 principal caudal‐fin rays. The procurrent rays are not clearly recognizable.

### Median fins

The origin of both the dorsal and anal fins is located at about mid‐length of the body (Fig. 2). The dorsal fin is large, nearly triangular and contains about 16 distally segmented rays decreasing in length posteriorly in series (Fig. 4A). The dorsal‐fin rays are supported by an equal number of pterygiophores, interdigitating with the neural spines of vertebrae 5–20. The anal fin is long‐based, more extended than the dorsal one, and contains about 25 rays supported by a similar number of pterygiophores. These latter elements interdigitate with the haemal spines of vertebrae 13–24.

### Paired fins and girdles

The pectoral girdle (Figs 2, 3) is consistent with that of most paraclupeid fishes (Alvarado‐Ortega *et al*. 2008; Murray & Wilson 2013). The posttemporal is thin, with a slender and sharp dorsal process. The supracleithrum is shorter than the posttemporal, considerably thick and slightly curved. The cleithrum is sigmoid in shape and represents the largest element of the pectoral girdle. The laminar coracoid is poorly ossified, broad and deep. The scapula is difficult to recognize. There is no evidence of the postcleithra. The pectoral fin seems to contain about ten rays, although the original number was probably higher.

The pelvic fins originate behind the sixth prepelvic scute (Figs 2, 4C). The number of pelvic‐fin rays is unclear. The basipterygia are only partially exposed and appear to be small and subtriangular in shape.

### Dorsal and ventral scutes

There is a complete series of at least 6 (or 7) predorsal scutes extending from the posterior edge of the skull roof to the dorsal‐fin origin (Fig. 4A). Some of the scutes appear to be ornamented with a few grooves. The anteriormost scutes are small, not significantly broader than long. The scutes increase in size posteriorly in the series and the posteriormost scutes bear a strong and prominent median spine. There are no serrations along the posterior margin of predorsal scutes. Postdorsal scutes are absent. The abdominal scute series is complete and extends from the isthmus to the anal‐fin origin. Their lateral wings are spine‐like, with large spaces between wings of adjacent scutes, and extend upward covering the abdominal cavity laterally for more than one‐third of the distance from the ventral margin of the body to the vertebral column. There are about 12 keeled abdominal scutes, including 6 prepelvic and 6 postpelvic elements decreasing in size posteriorly. The postpelvic scutes bear a very prominent and strong ventral posteriorly‐directed spine (Fig. 4C).

## Phylogenetic analysis

**Figure 6:**
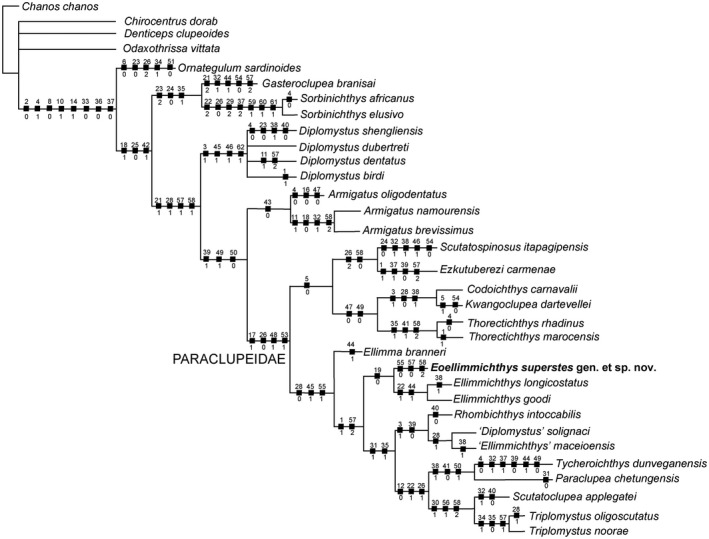
The strict consensus tree retrieved in TNT 1.5 based on 62 morphological characters and 33 taxa, showing the hypothetic phylogenetic relationship of *Eoellimmichthys superstes* gen. et sp. nov. within the Ellimmichthyiformes. Number character above and state below on each node.

The analysis of 62 morphological characters coded for 32 taxa (see Marramà *et al*. 2018a) produced 10 equally parsimonious trees that were used to build a single strict consensus tree with a tree length of 199 steps (CI = 0.38; RI = 0.62; Fig. 6). The relationships among ellimmichthyiforms are highly consistent with those resulting from the analysis conducted by Vernygora & Murray (2015) and Marramà & Carnevale (2017a). In our study, the monophyly of the Ellimmichthyiformes is supported by eight synapomorphies for which state ‘0’ may not always be the basal condition for clupeomorphs: parietals contacting each other in the midline (ch. 2[0]); ornamentation of skull roof bones present (ch. 4[1]); recessus lateralis absent (ch. 8[0]); ‘basipterygoid’ process of parasphenoid present (ch. 10[1]); beryciform foramen on anterior ceratohyal (ch. 14[1]); first uroneural and first ural centrum not fused (ch. 33[0]); parhypural fused to first preural centrum (ch. 36[0]); three epurals (ch. 37[0]). The monophyletic status of *Gasteroclupea *+ Sorbinichthyidae is supported by the three characters presented by Marramà & Carnevale (2017a). As revealed in the study by Vernygora & Murray (2015), the genus *Diplomystus* (with the exclusion of the paraclupeid ‘*D*.’ *solignaci*) should be considered monophyletic and represents the sister‐group of the pair formed by the families Armigatidae and Paraclupeidae. However, the relationships between the valid species of the genus *Diplomystus* remain unresolved. The monophyly of the family Paraclupeidae is supported in our study by four characters: halves of the neural arches of most abdominal vertebrae fused medially (ch. 17[1]); proximal end of first hypural massive and forming an upward process (ch. 26[0]); predorsal scutes increasing in size posteriorly (ch. 48[1]); postpelvic abdominal scutes bearing a very prominent and strong ventral spine (ch. 53[1]). Our study reveals a dichotomous nature of Paraclupeidae in which some taxa (*Codoichthys*,* Scutatuspinosus*,* Ezkutuberezi*,* Kwangoclupea* and *Thorectichthys*) are characterized by ornamented skull roof bones with fine, and more or less parallel grooves (ch. 5[0]), whereas all the remaining paraclupeids share a third hypural not expanded posteriorly leaving a gap or notch between the second and third hypurals (ch. 28[0]); subrectangular scutes in the posterior part of predorsal series (ch. 45[1]); and wide or spatulate lateral wings of abdominal scutes, with wings of adjacent scutes contacting each other for most of their length (ch. 55[1]). This last character is reversed in *Eoellimmichthys*, which has wings spine‐like, with large spaces between wings of adjacent scutes (ch. 55[0]). The Early Cretaceous *Codoichthys* from north‐east Brazil was not recovered as the basalmost paraclupeid as suggested by Figueiredo & Ribeiro (2016); rather, our phylogeny recovers a sister‐group relationship with the Late Cretaceous *Kwangoclupea* from Zaire, with which it shares at least three synapomorphies: lateral profile of the skull roof with distinct angle between anterior and posterior parts (ch. 3[1]); third hypural not expanded posteriorly leaving a gap or notch between the second and third hypurals (ch. 28[0]); epurals located far from the spine of the second preural vertebra, leaving an open space between them (ch. 38[1]). The phylogenetic placement of *Eoellimmichthys* within Paraclupeidae is clearly evidenced in our results (Fig. 6). *Eoellimmichthys* and *Ellimmichthys* form a monophyletic clade sharing a single character, absence of epineurals and epipleurals in the caudal region (ch. 19[0]). The clade formed by these two genera represents the sister group of the more derived paraclupeids, which were included by Murray & Wilson (2013) in the subfamilies Ellimmichthyinae (*Rhombichthys intoccabilis* + (‘*Ellimmichthys*’ *maceioensis* + ‘*Diplomystus*’ *solignaci*)) and the Paraclupeinae ((*Paraclupea chetungensis* + *Tycheroichthys dunveganensis*) + (*Scutatuclupea applegatei* + (*Triplomystus oligoscutatus* + *Triplomystus noorae*))). The sister‐group relationship of the two species of *Ellimmichthys* is supported in our study by two characters, which are absent in *Eoellimmichthys*: dorsal process of the posttemporal subrectangular (ch. 22[1]); and subrectangular scutes significantly broader than long also in anterior part of predorsal series (ch. 44[1]). Finally, the pair *Eoellimmichthys* + *Ellimmichthys* shares at least two synapomorphies with the most derived paraclupeids: anterior dorsal margin of the body almost straight, forming a marked angle at the level of the dorsal‐fin insertion (ch. 1[1]) and more than 32 abdominal scutes (ch. 57[2]); this latter feature exhibits a reversal in *Eoellimmichthys* (fewer than 20; ch. 57[0]).

## Discussion

### Comparative remarks

The morphological analysis of *Eoellimmichthys* gen. nov. has revealed the presence of a series of characters justifying its inclusion within the Clupeomorpha, including the presence of one or more abdominal scutes crossing the ventral midline of the body, and dorsal scutes with a median keel. Other clupeomorph synapomorphies (e.g. otophysic connection that penetrates the exoccipital and forming an ossified bulla in the prootic and usually also in the pterotic; supratemporal commissural sensory canal primitively passing through parietals; presence of a well‐defined pre‐epiotic fossa; autogenous first hypural; Grande 1985) cannot be determined in the single available specimen due to its inadequate preservation. The affinities of *Eoellimmichthys* with the members of the order Ellimmichthyiformes are supported by the presence of ornamentation of the skull bones, medial fusion of the contralateral halves of the neural arches in most abdominal vertebrae, endopterygoid teeth, and a parhypural that is fused to the first preural centrum (Alvarado‐Ortega *et al*. 2008; Murray & Wilson 2013; Vernygora & Murray 2015); however, some of these features have been considered as plesiomorphic for clupeomorphs by some authors (Patterson & Rosen 1977; Grande 1982, 1985; Arratia 1997). *Eoellimmichthys* exhibits a suite of features that clearly define its separate status among ellimmichthyiforms. In particular, this new genus is characterized by a complete series of 6 (or 7) predorsal scutes extending from the occiput to the dorsal‐fin origin. For this reason, it can be easily separated from *Armigatus*, which shows an incomplete series of predorsal scutes leaving a gap just posterior of the occiput (Grande 1982; Forey *et al*. 2003; Vernygora & Murray 2015). *Eoellimmichthys* cannot be included within the Sorbinichthyoidei since the representatives of this suborder are characterized by the presence of 10 or more supraneurals, and more than 20 predorsal scutes. The alignment of *Eoellimmichthys* with the genus *Diplomystus* can be ruled out because the latter genus shows subrectangular scutes with a posterior margin that is ornamented with several small spines, and a fan‐shaped arrangement of the supraneurals (Grande 1982; Chang & Maisey 2003; Vernygora & Murray 2015; Murray *et al*. 2016).

On the other hand the attribution of *Eoellimmichthys* to the family Paraclupeidae is clearly demonstrated by the presence of a short series of 6 (or 7) irregular predorsal scutes increasing in size posteriorly in the series, and postpelvic scutes bearing very prominent spines (Chang & Grande 1997; Forey *et al*. 2003; Alvarado‐Ortega *et al*. 2008; Khalloufi *et al*. 2010; Murray & Wilson 2013). Although the phylogenetic analysis recognized *Eoellimmichthys* as more closely related to the *Ellimmichthys* species from the Early Cretaceous of Brazil and western Africa than to other paraclupeids, its separate status within this clade can be easily recognized based on the absence of a subrectangular dorsal process of the posttemporal as well as of subrectangular scutes also in the anterior part of the predorsal series, which instead characterize the Early Cretaceous taxa (Grande 1982; Chang & Grande 1997). Moreover, *Eoellimmichthys* differs from the other paraclupeid genera in having a unique series of reductive meristic features (Table 1). In particular, both the number of abdominal scutes (12) and preural vertebrae (31) exhibit the lowest values within the family Paraclupeidae. Moreover, the new genus can be easily separated from *Codoichthys*,* Ellimma*,* Ellimmichthys*,* Ezkutuberezi*,* Paraclupea*,* Rhombichthys*,* Scutatuspinosus* and *Tycheroichthys* by the lower number of supraneurals (6 vs 7–12), and from all the paraclupeids (except *Ezkutuberezi* and *Kwangoclupea*) by the low number of predorsal scutes (6–7 vs 8–17) and pleural ribs (10 vs 13–25). Furthermore, *Eoellimmichthys* lacks the postdorsal scutes that are diagnostic of *Scutatoclupea* and *Triplomystus*. Finally, the number of anal‐fin pterygiophores separates *Eoellimmichthys* (25) from all the other paraclupeids, except *Thorectichthys*.

**Table 1:** Selected morphological features used to discriminate genera of the family Paraclupeidae

	Supraneurals	Predorsal scutes	Postdorsal scutes	Abdominal scutes	Pleural ribs	Preural vertebrae	Ribs/vertebrae ratio	Dorsal‐fin pterygiophores	Anal‐fin pterygiophores
*Codoichthys*	7	8	0	20	17	33	0.52	10	15
*Ellimma*	8	12–14	0	27–30	20–22	36–38	0.56–0.58	14–16	14–15
*Ellimmichthys*	8–9	12–13	0	32–35	20–22	35–36	0.56–0.63	12–15	9–13
***Eoellimmichthys***	**6**	**6**–**7**	**0**	**12**	**10**	**31**	**0.32**	**16**	**25**
*Ezkutuberezi*	12	5+	0	30	13–14	37	0.37	24	23
*Kwangoclupea*	7	6–7	0	30	10–11	32	0.33	11–13	27–29
*Paraclupea*	7–9	18	0	33–43	20–23	38–41	0.52–0.56	16–18	13–17
*Rhombichthys*	7–8	12	0	35–46	20	34	0.59	26	12–13
*Scutatoclupea*	6–9	13–17	21–22	33–42	?	39–44	?	18–19	12–16
*Scutatuspinosus*	10	10	0	25	17	32	0.53	10	8
*Thorectichthys*	5–6	8–11	0	22–26	14–18	33–38	0.42–0.47	14–17	22–25
*Triplomystus*	5–6	12–13	10–12	24–33	15–20	36	0.43–0.56	16–20	16–24
*Tycheroichthys*	7	16	0	36	25	40	0.62	19	23

? Missing data.

Table includes new data and data from Grande (1982, 1985), Chang & Grande (1997), Taverne (1997), Poyato‐Ariza *et al*. (2000), Chang & Maisey (2003), Forey *et al*. (2003), Hay *et al*. (2007), Alvarado‐Ortega & Ovalles‐Damián (2008), Khalloufi *et al*. (2010), Murray & Wilson (2013), Bannikov (2015), Figueiredo & Ribeiro (2016, 2017), and Alvarado‐Ortega & Melgarejo‐Damián (2017).

### Evolutionary and biogeographical observations

The Ellimmichthyiformes have a long evolutionary history encompassing about 90 million years from the Early Cretaceous to the middle Eocene (Grande 1982, 1985). Figueiredo & Ribeiro (2017) considered the paraclupeid *Scutatuspinosus itapagipensis* from the Hauterivian–Barremian Marfim Formation, Recôncavo Basin, Brazil, to be the oldest confirmed double‐armoured herring. Another paraclupeid, *Ellimmichthys longicostatus*, occurs along with *Scutatuspinosus itapagipensis* in the Marfim Formation of Brazil (e.g. Chang & Maisey 2003) thereby extending back, to the earliest part of the Cretaceous, the age of divergence between armigatids and paraclupeids and, more generally, the age of appearance of the double‐armoured herrings. However, *Ezkutuberezi carmenae* might be older than the Brazilian taxa since it has been recovered from freshwater deposits of Valanginian–Barremian age (Poyato‐Ariza *et al*. 2000). Other basal clupeomorphs that seem to pertain to the clade Ellimmichthyiformes might be older than these Early Cretaceous Brazilian or Iberian fossils. Some of these ancient double‐armoured herrings were referred to the ‘waste‐basket’ genera ‘*Clupea*’ or ‘*Diplomystus*’ following the common practice of most authors from the nineteenth and twentieth centuries, and it is likely that taxonomic revision will demonstrate that they do not belong to these taxa (Grande 1985). For example, the double‐armoured herrings ‘*Diplomystus*’ *altisomus*, ‘*D*.’ *kokuraensis*, and ‘*D*.’ *primotinus* from the lacustrine ‘Neocomian’ deposits of Japan were assigned to *Diplomystus* by Uyeno (1979) and Yabumoto (1994), based on supposed similarities with this extinct genus whose diagnostic characters have been properly defined only more recently by Chang & Maisey (2003). Pictet (1858) described ‘*Clupea*’ *antiqua* and ‘*C*.’ *voironensis* based on some specimens from the Early Cretaceous of Switzerland, and considered them to be closely related to modern sardines of the family Clupeidae, although they are probably related to *Armigatus*.

Double‐armoured herrings experienced a remarkable increase in species diversity during the middle and Late Cretaceous that was probably mirrored by a considerable increase of their morphological diversity, exemplified by the appearance of certain peculiar and extreme shapes, very different from the basic herring or sardine‐like shape of most clupeomorphs. For example, Late Cretaceous (mostly Cenomanian) taxa such as *Tycheroichthys dunveganensis*,* Triplomystus noorae*, and *Rhombichthys intoccabilis* were characterized by extremely deep bodies, large and deeply forked caudal fins, as well as by a strong armour consisting of long and well‐developed abdominal keeled scutes (Forey *et al*. 2003; Hay *et al*. 2007; Khalloufi *et al*. 2010). The order Ellimmichthyiformes was not the only fish group that experienced a steep increase in disparity and morphological adaptations in the Late Cretaceous. Starting in the Cenomanian a number of actinopterygian lineages, including among others the pycnodonts and acanthomorphs, also evolved a remarkable range of adaptations and morphologies (Tyler & Sorbini 1996; Marramà *et al*. 2016d), with the proliferation of new bauplans and, at least in part, the exploitation of new ecological strategies and anti‐predatory solutions (Poyato‐Ariza 2005; Marramà *et al*. 2016d) possibly in response to the large predator–prey escalation in the context of the Mesozoic Marine Revolution (Chen *et al*. 2014; Marramà *et al*. 2016d). The Cretaceous diversification of double‐armoured herrings was possibly related to major changes in abundance and composition that affected the marine plankton (Leckie *et al*. 2002) after the middle Cretaceous, allowing these planktivorous fishes to exploit new habitats and trophic resources. Ellimmichthyiforms successfully occupied the intermediate trophic levels of the marine food web during most of the Cretaceous (Cavin 2002) and the end‐Cretaceous mass extinction event resulted in marked intensive faunal changes that also strongly affected ellimmichthyiform diversity, from both a taxonomic and an ecological point of view. The decline of primary productivity, which involved all trophic levels of the food web, including the intermediate levels occupied primarily by clupeomorphs, has been considered to be the most likely cause of extinction in the marine domain (Ware & Thomson 2005; D'Hondt 2006; Robertson *et al*. 2013; Bardeen *et al*. 2017). By the end of the Cretaceous, and more extensively since the early Paleocene, modern clupeiforms were characterized by a vast morphological diversification in the marine domain through which these fishes filled the ecological niche left vacant by the double‐armoured herrings and other planktivorous taxa. Such a massive diversification was also characterized by the appearance of piscivorous clupeiforms of the wolf herring lineage (Chirocentridae), which participated to the occupation of the vacant niches left open by the large pelagic predatory taxa victims of the end‐Cretaceous extinction (Cavin 2002; Friedman 2009, 2010; Friedman & Sallan 2012).

The Paleocene fish fauna is generally characterized by end‐Cretaceous mass extinction survivors and double‐armoured herrings have been regarded as a relict lineage that exclusively persisted in freshwater environments up to the middle Eocene (Grande 1982, 1985). The freshwater environments were extremely resilient and largely avoided a mass extinction at the end of the Cretaceous, acting as refuges for several ancient fish lineages such as amiiforms and lepisosteiforms that, like the ellimmichthyiforms, were affected by the complete disappearance of marine forms at the Cretaceous–Palaeogene boundary (Cavin 2002). In this context, the Cenozoic record of double‐armoured herrings was considered to be exclusively restricted to few freshwater taxa, including the peculiar sorbinichthyioid *Gasteroclupea branisai* from the Upper Cretaceous to Paleocene freshwater deposits of South America (Marramà & Carnevale 2017a), and *Diplomystus dentatus* and *D. shengliensis* that were confined to the middle Eocene freshwaters of North America and China, respectively (Grande 1982; Chang & Maisey 2003).

The Bolca bony fish assemblage includes some of the youngest occurrences of extinct Mesozoic lineages, including the Pycnodontiformes, represented therein by four taxa, which are the last representatives of this non‐teleost actinopterygian clade in a marine setting (Blot 1987; Poyato‐Ariza & Wenz 2002; Carnevale *et al*. 2014). Other relicts among bony fishes of Bolca are the pachyrhizodontoids, with the conspicuous *Platinx macropterus*, which possibly represents the youngest occurrence of the order Crossognathiformes, a lineage of Late Jurassic to Cretaceous marine basal teleosts (e.g. Taverne 1980; Cavin 2001; Arratia 2008). Consequently, *Eoellimmichthys* along with the sympatric pycnodontiforms and crossognathiforms, has to be regarded as a genuine survivor of the end‐Cretaceous extinction in the marine realm. Although the role of the shallow waters of the Western Tethys, including those of the Bolca area, were recognized as primary hotspot of biodiversity during the early Palaeogene with increased origination rates in the aftermath of the K/Pg boundary event (see Renema *et al*. 2008), this region might also have acted as marine refuge for the last survivors of several actinopterygian lineages, such as pycnodontiforms, that were highly diverse before the end‐Cretaceous mass extinction event and ultimately vanished before the end of the Eocene. Interestingly, pycnodontiforms were still widespread in the Eocene from the eastern coastline of the North America in the West to India in the east and Africa in the south, including freshwater occurrences (e.g. Longbottom 1984). Probably, pycnodontiforms paralleled ellimmichthyiforms in that they persisted largely unscathed in freshwaters during the end‐Cretaceous event, but were affected in marine environments (Kriwet 2001a, 2001b). The reasons for the final disappearance of various actinopterygian clades in the Eocene are still ambiguous and might be related to competition (e.g. in pycnodontiforms; Bellwood 1996) or climatic perturbations (Eocene–Oligocene climatic cooling event).

**Figure 7:**
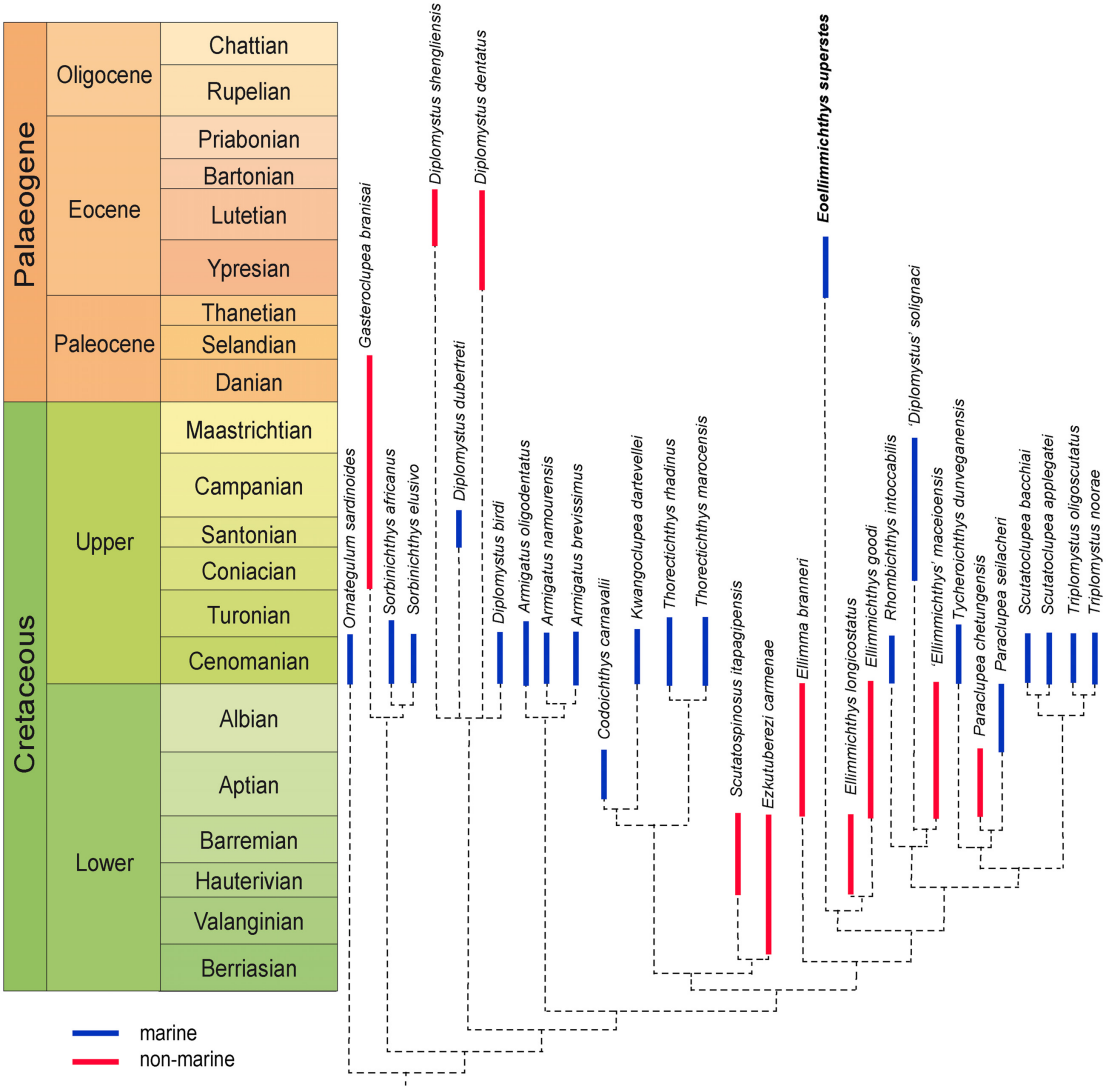
Time‐calibrated phylogeny of the Ellimmichthyiformes illustrating the major divergences of subgroups of double‐armoured herrings based on fossil evidence. Blue lines are used to indicate marine taxa, whereas red lines are used to indicate non‐marine taxa. Data from Grande (1982, 1985), Chang & Grande (1997), Taverne (1997), Chang & Maisey (2003), Forey *et al*. (2003), Hay *et al*. (2007), Alvarado‐Ortega & Ovalles‐Damián (2008), Khalloufi *et al*. (2010), Poyato‐Ariza *et al*. (2000), Murray & Wilson (2013), Bannikov (2015), Vernygora & Murray (2015), Figueiredo & Ribeiro (2016, 2017), Murray *et al*. (2016), Alvarado‐Ortega & Melgarejo‐Damián (2017) and Marramà & Carnevale (2017a). Chronostratigraphic scheme adopted and modified from International Chronostratigraphic Chart v 2016/12. Colour online.

*Eoellimmichthys* considerably increases our knowledge of the evolutionary history of double‐armoured herrings, extending the stratigraphic range of the family Paraclupeidae up to the Ypresian (Fig. 7). To date, the poorly known ‘*Diplomystus*’ *solignaci* from the Senonian of El Hamma de l'Arad, Tunisia (Gaudant & Gaudant 1971) was considered to be the youngest member of this family, whose earliest members likely appeared in the Hauterivian or Barremian (e.g. Chang & Maisey 2003). The long evolutionary history of this family was associated with the occupation of a variety of habitats and a considerably wide geographical distribution (Fig. 7). The majority of the Early Cretaceous paraclupeids was restricted to freshwater sediments of the Western Gondwana rift basins (e.g. *Ellima branneri*,* Ellimmichthys goodi*,* E. longicostatus*, ‘*Ellimmichthys*’ *maceioensis*,* Scutatuspinosus itapagipensis*; see Chang & Maisey 2003, Malabarba *et al*. 2004, Figueiredo & Ribeiro 2017), Iberia (*Ezkutuberezi carmenae*; Poyato‐Ariza *et al*. 2000) and China (*Paraclupea chetungensis*; Chang & Grande 1997), but some taxa are also known from shallow marine deposits of Brazil and Mexico (*Codoichthys carnavalii*,* Paraclupea seilacheri*,* Scutatoclupea applegatei*; see Alvarado‐Ortega & Ovalles‐Damián 2008; Figueiredo & Ribeiro 2016; Alvarado‐Ortega & Melgarejo‐Damián 2017). Vernygora *et al*. (2016) discussed the freshwater and/or estuarine affinities of the earliest known paraclupeids, and more generally of double‐armoured herrings, suggesting that the analysis of this distributional pattern might reveal new insights into the origin of the diadromy, also providing information on the evolution of the osmoregulatory system in clupeomorph fishes. Although the distribution pattern of certain freshwater taxa, as for example that of the sister pair formed by *Ellimmichthys goodi* (late Aptian – Albian of Equatorial Guinea) and *E. longicostatus* (Hauterivian–Barremian of Brazil), provides clear evidence of vicariance between both sides of the South Atlantic (see Maisey 2000), no biogeographical hypothesis satisfactorily explains the known overall distribution of Early Cretaceous freshwater paraclupeids (Chang & Maisey 2003). A number of marine paraclupeids are known from the Cenomanian of the Tethys (*Rhombichthys intoccabilis*,* Scutatoclupea bacchiai*,* Thorectichthys marocensis*,* T. rhadinus*,* Triplomystus oligoscutatus*,* T. noorae*; see Forey *et al*. 2003; Khalloufi *et al*. 2010; Murray & Wilson 2013; Bannikov 2015), eastern South Atlantic (*Kwangoclupea dartevellei*; Taverne 1997) and northern part of the Western Interior Seaway (*Tycheroichthys dunveganensis*; Hay *et al*. 2007), revealing that the global distribution of the group is possibly related to the high global temperatures and sea level stand characteristic of that period (e.g. Gale 2000). As suggested by Murray & Wilson (2013), the sister group relationship between *Paraclupea* and *Tycheroichthys* (reiterated by our phylogenetic analysis) suggests that paraclupeids at least were capable of long‐distance dispersal. In any case, the biogeographical history of paraclupeids is difficult to interpret due to our inadequate knowledge of their fossil record. The unexpected Eocene marine paraclupeid described herein clearly demonstrates that additional fossil finds are necessary to achieve a better understanding of the evolutionary history and biogeography of double‐armoured herrings.

## Conclusions

The morphological and phylogenetic analysis of a nearly complete articulated clupeomorph has revealed for the first time the presence of a representative of the clupeomorph order Ellimmichthyiformes in the early Eocene limestone of the Bolca Konservat‐Lagerstätte. The presence of clupeiforms and ellimmichthyiforms in the Pesciara site provides evidence of the heterogeneity of the clupeomorphs of this celebrated locality, reflecting the outstanding diversity of the entire fish assemblage (Carnevale *et al*. 2014). This youngest marine record of the double‐armoured herrings provides further evidence that fish lineages which were severely affected by the end‐Cretaceous extinction (ellimmichthyiforms, pycnodontiforms and crossognathiforms) survived at least until the late Ypresian in tropical shallow marine palaeobiotopes of the Tethys which might have acted as a refuge.
